# *Bacillus sphaericus* exposure reduced vector competence of *Anopheles dirus* to *Plasmodium yoelii* by upregulating the Imd signaling pathway

**DOI:** 10.1186/s13071-020-04321-w

**Published:** 2020-09-05

**Authors:** Shasha Yu, Pan Wang, Jie Qin, Hong Zheng, Jing Wang, Tingting Liu, Xuesen Yang, Ying Wang

**Affiliations:** 1 Department of Tropical Medicine, College of Military Preventive Medicine, Army Medical University, Chongqing, 400038 China; 2 Department of Thoracic Surgery, Xinqiao Hospital, Army Medical University, Chongqing, 400037 China

**Keywords:** *Bacillus sphaericus*, *Anopheles dirus*, *Plasmodium yoelii*, Vector competence, Malaria, TEP1, Imd signaling pathway

## Abstract

**Background:**

Vector control with *Bacillus sphaericus* (Bs) is an effective way to block the transmission of malaria. However, in practical application of Bs agents, a sublethal dose effect was often caused by insufficient dosing, and it is little known whether the Bs exposure would affect the surviving mosquitoes’ vector capacity to malaria.

**Methods:**

A sublethal dose of the Bs 2362 strain was administrated to the early fourth-instar larvae of *Anopheles dirus* to simulate shortage use of Bs in field circumstance. To determine vector competence, mosquitoes were dissected and the oocysts in the midguts were examined on day 9–11 post-infection with *Plasmodium yoelii*. Meanwhile, a SYBR quantitative PCR assay was conducted to examine the transcriptional level of the key immune molecules of mosquitoes, and RNA interference was utilized to validate the role of key immune effector molecule TEP1.

**Results:**

The sublethal dose of Bs treatment significantly reduced susceptibility of *An. dirus* to *P. yoelii*, with the decrease of *P. yoelii* infection intensity and rate. Although there existed a melanization response of adult *An. dirus* following challenge with *P. yoelii*, it was not involved in the decrease of vector competence as no significant difference of melanization rates and densities between the control and Bs groups was found. Further studies showed that Bs treatment significantly increased TEP1 expression in the fourth-instar larvae (L4), pupae (Pu), 48 h post-infection (hpi) and 72 hpi (*P* < 0.001). Further, gene-silencing of TEP1 resulted in disappearance of the Bs impact on vector competence of *An. dirus* to *P. yoelii*. Moreover, the transcriptional level of PGRP-LC and Rel2 were significantly elevated by Bs treatment with decreased expression of the negative regulator Caspar at 48 hpi, which implied that the Imd signaling pathway was upregulated by Bs exposure.

**Conclusions:**

Bs exposure can reduce the vector competence of *An. dirus* to malaria parasites through upregulating Imd signaling pathway and enhancing the expression of TEP1. The data could not only help us to understand the impact and mechanism of Bs exposure on *Anopheles*’ vector competence to malaria but also provide us with novel clues for wiping out malaria using vector control.
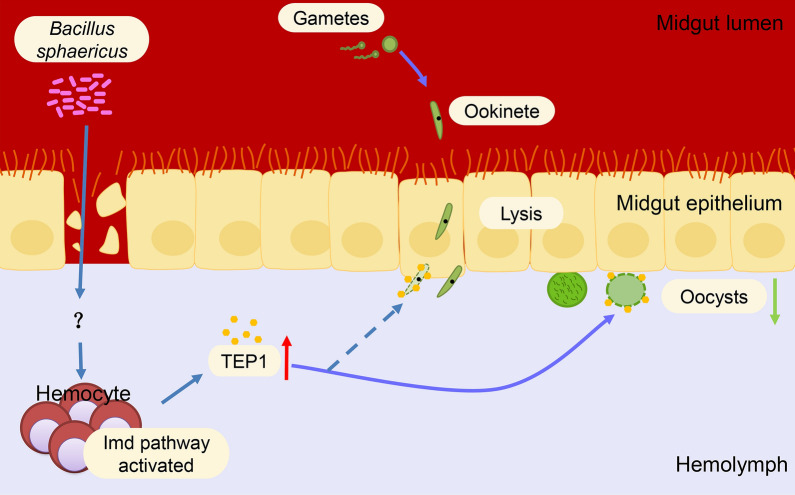

## Background

Malaria which is transmitted by *Anopheles* mosquitoes is still one of the most devastating disease worldwide, causing 228 million infections and approximately 405,000 deaths in 2018 [1]. Vector control is recognized as an effective way to control this disease [2]. Chemical insecticides have been used for several decades to control mosquitoes. However, the extensive and intensive use of chemical insecticides has been greatly impeded due to development of physiological resistance in the vectors, environmental pollution resulting in bio-amplification of food chain contamination and undesirable effects on beneficial organisms [3, 4]. Therefore, the need for alternative, more effective and environment-friendly control agents becomes urgent.

*Bacillus sphaericus* (Bs) has been used as an ideal mosquito biolarvicide and put into mass production because of its good biological effects [5]. Bs is an aerobic, rod-shaped, endospore-forming, gram-positive soil bacterium, which was firstly reported toxic to mosquito larvae by Kellen et al. in 1965 [6]. The mosquito larvicidal activity of Bs is attributed mainly to binary toxins in most of highly toxic strains and Mtx toxins in low toxic strains. In target insect species, the crystal proteins can be activated by the mosquito midgut protease and become poisonous polypeptides, then bind to receptors of midgut epithelial cells and result in epithelial cell perforations and death of larvae [7, 8]. Compared with other biological insecticides, Bs has several advantages, including low environmental toxicity due to the high specificity of Bs toxins, high levels of efficacy, and environmental persistence. Meanwhile, the use of Bs recombination agents can also extend the mosquito-killing spectrum, prolong the action time, and improve the insecticidal efficacy [9–11]. Furthermore, it was reported that Bs successfully decreased the spread of mosquito-borne diseases in epidemic areas [12–14]. Hence, *B. sphaericus* is attracting more and more attention, and has been considered as one of the most economical and efficient biolarvicide.

However, the fundamental research of Bs application is limited up to now, especially on the subsequent effect of Bs use. In practical terms, a sublethal dose effect often existed because of the insufficient dosing and led to some mosquitoes’ survival. Obviously, the sublethal dose effect exerts a selection pressure on the surviving mosquito populations and might change their physiological functions. It has been reported that bacterial exposure at the larval stage would induce immune priming in adult *Aedes aegypti* mosquitoes [15, 16], which would finally affect the vector competence of mosquito-borne diseases, such as dengue virus. Whether the sublethal dose of Bs treatment would change the vector competence of *Plasmodium* to *Anopheles* mosquitoes by eliciting the immune priming remains unknown. *Anopheles dirus* is one of the most important vectors transmitting malaria in China and Southeast Asia [17, 18]. In this study, we focused on exploring whether the sublethal dose of Bs (SdBs) will affect vector competence of *An. dirus* to malaria, and discuss the interaction mechanism between SdBs and *Anopheles* mosquitoes. It will be helpful to develop new and better ways in the fight against malaria.

## Methods

### *Bacillus sphaericus*, mice and *Plasmodium* parasite

*Bacillus sphaericus* (Bs) 2362 strain was provided by Hubei Kangxin agricultural pharmaceutical company, and the titer of Bs was 650 IU/mg. Kunming outbred Swiss Webster mice were bought from the Experimental Animal Center of the Army Medical University, Chongqing, China. *Plasmodium yoelii* red fluorescence protein transgenic BY265 strain (*P. yoelii* BY265 RFP) was kindly donated by Professor Wenyue Xu from the Army Medical University, and maintained by passage between Kunming mice and mosquitos.

### Mosquito larva rearing and sublethal dose of Bs treatment

*Anopheles dirus* (Hainan strain) larvae were fed on a powder mix of dry degreased pork liver and yeast at a ratio of 1:4 at 28 °C with a 12 h light/dark cycle. A sublethal dose of the Bs 2362 strain was administrated to the early fourth-instar larvae for 48–72 h to simulate field conditions with shortage use of Bs, according to the bioassay results of the previous research in our laboratory [19]. When the surviving larvae developed into pupae, they were collected and further emerged to adults in cages. The adult mosquitoes were then routinely maintained under the same environmental and rearing conditions as the group without Bs treatment.

### Mosquito adult rearing and infection

The Bs-treated and normal control adult mosquitoes were maintained at 28 °C, 70–80% relative humidity and fed on 10% sugar solution with a 12 h light/dark photocycle, according to standard rearing procedures in the laboratory. For infection with *P. yoelii* BY265 RFP, 3- to 5-day-old female adults were removed and kept at 24 °C and fed on *yoelii* BY265 RFP-infected Kunming mice with a gametocytemia above 0.5%. On day 9–11 post-infection, mosquitoes were dissected, and the oocysts in the midguts were examined under a fluorescence microscope. The infection rate and the intensity of *Plasmodium* were then calculated as well as the intensity and the rate of melanization.

### RNA extraction and cDNA synthesis

Total RNA was extracted from ~10 female mosquitoes of each group using TRIzol (Invitrogen, Carlsbad, CA, USA) as per the manufacturer’s protocol, after pulverizing the tissues in a homogeniser on ice. Total RNA was finally dissolved in 40 µl RNase-free water and 1 µg total RNA was reverse transcribed to cDNA in accordance with the instructions of the reverse transcription kit (Takara, Dalian, Liaoning, China).

### Real-time PCR

Real-time quantitative PCR was conducted to examine the transcriptional level of the key immune molecules of mosquitoes such as TEP1, PGRP-LC, PGRP-S1, Rel2, Caspar, MyD88, Tube, Rel1, STAT and PIAS2. The conserved S7 of *An. dirus* was used as the reference gene. The PCR reactions (20 μl) contained 0.4 μl of each primer (10 μM), 10 μl KAPA SYBR® FAST qPCR Kit Master Mix 2× Universal (KAPA Biosystems, Wilmington, MA, USA), 8.2 μl ddH_2_O and 1 μl cDNA. Real-time PCR was performed on a Bio-Rad CFX96 Touch™ real-time PCR instrument equipped with a 96-well reaction block (Bio-Rad, Hercules, California, USA). The SYBR Green quantitative PCR cycling conditions consisted of an initial denaturation at 95 °C for 3 min followed by amplification for 40 cycles of 3 s at 95 °C and 30 s at 60 °C, and a melt curve step from 65 to 95 °C, with increment of 0.5 °C every 5 s. The expression of each gene relative to ribosomal S7 RNA was determined using the 2^−ΔΔCT^ method [20]. The primers are listed in Table 1 and Additional file 1: Table S1.

**Table 1:** Gene primers for real-time PCR

Gene	Forward primer (5′-3′)	Reverse primer (5′-3′)
Ad TEP1	TGGAGCATCAGGGTTCTAGG	TGGACAGGTCGTAAGGTT
Ad PGRP-LC	ACCAGGGGCAGTCTCTCCAATATC	CATCATCGCACTCGGTATCGCTAC
Ad PGRP-S1	ACAACTGCTGCTGCTGGTTCTG	CGCCTCACAATGGTCGGACAG
Ad Rel2	TGACCACAGTGCATCGTACA	GTTGCTGCTGCTGCTGATAG
Ad Caspar	AACAACAGCCACAGCAGCAGTAG	GTCCGACGCATCCTCAAACTCTTC
Ad S7	CAACAACAAGAAGGCGATCA	GACGTGCTTACCGGAGAACT

### RNA interference

RNA interference was carried out to confirm the function of TEP1 in changing of susceptibility of *An. dirus* to *P. yoelii* infection by Bs exposure. To synthesize the TEP1 double-stranded RNA (dsRNA), *An. dirus* cDNA was subjected to PCR using gene-specific primers with a 5′ extension of T7 promoter tags (5′-TAA TAC GAC TCA CTA TAG GG-3′). The control dsRNA was made from a fragment of the GFP gene (see Table 2). The PCR products were purified and used as templates for *in vitro* transcription reactions with an Ambion Megascript RNAi kit (Ambion Life Technologies, Austin, TX, USA), following the manufacturer’s instructions. The resulting dsRNA was analyzed by agarose gel electrophoresis and the concentrations were determined using BioTek Epoch (Biotek, Winooski, Vermont, USA). Approximately 69 nl dsRNA (2–3 mg/ml) of TEP1 was introduced into the thorax of ether-anesthetized 2–4-day-old female mosquitoes by a nano-injector (Drummond Scientific Co., Bromall, PA, USA). The mortality caused by injection was about 20% with no significant difference among groups.

**Table 2:** Specific gene primers used in RNAi

Gene	Forward primer (5′-3′)	Reverse primer (5′-3′)
AdTEP1 ds RNA	TAATACGACTCACTATAGGGATCGGCTCGCTTCCAAACAAAGG	TAATACGACTCACTATAGGGTACTCTCTCAATGTTCTTCTCTG
GFP ds RNA	TAATACGACTCACTATAGGGTCAAGTTCAACGTGTCCGGCG	TAATACGACTCACTATAGGGAGGACCATTTGATCGCGCTT

Three days after dsRNA injection, the gene silencing efficiency was confirmed by semi-quantitative PCR and western blot. For western blotting experiments, proteins of 10–20 female mosquitoes of each group were extracted by Western and IP cell lysate (Beyotime, Shanghai, China) following the manufacturer’s “instructions for protein isolation” protocol. These samples were then separated on 10% SDS-PAGE gels and subsequently transferred to Hybond nitrocellulose membranes. The membranes were blocked with 5% skimmed milk for 1 h, and then incubated with anti-AdTEP1 antibody (previously prepared in our laboratory) at a 1:1000 dilution overnight at 4 °C. After three washes of 10 min in TBST, the membranes were incubated with anti-rabbit secondary antibody at a 1:2000 dilution for 1 h at room temperature. Three more washes were performed before the incubation of the membrane with the detection system Clarity Western ECL Substrate (Bio-Rad).

Meanwhile, after being treated or untreated with AdTEP1 or GFP dsRNA for 3 days, mosquitoes were fed on the same mouse infected with *P. yoelii* BY265 RFP. On day 9–11 post-infection, mosquitoes were dissected, and the oocysts in the midguts were counted under a fluorescence microscope. The infection and melanization rates and intensities were calculated after RNA interference both in the Bs and control groups.

### Statistical analysis

All statistical analyses were performed using IBM SPSS Statistic 19.0 (SPSS Inc., Chicago, IL, USA). The Chi-square test was used to compare the infection and melanization rates and the Mann-Whitney U-test was used for the data with non-normal distribution to compare the oocyst counts in Bs and control groups. *P*-values < 0.05 were considered statistically significant.

## Results

### Sublethal dose of Bs treatment significantly reduced the intensity and rate of *P. yoelii* infection in *An. dirus* while melanization was not affected

**Fig. 1:**
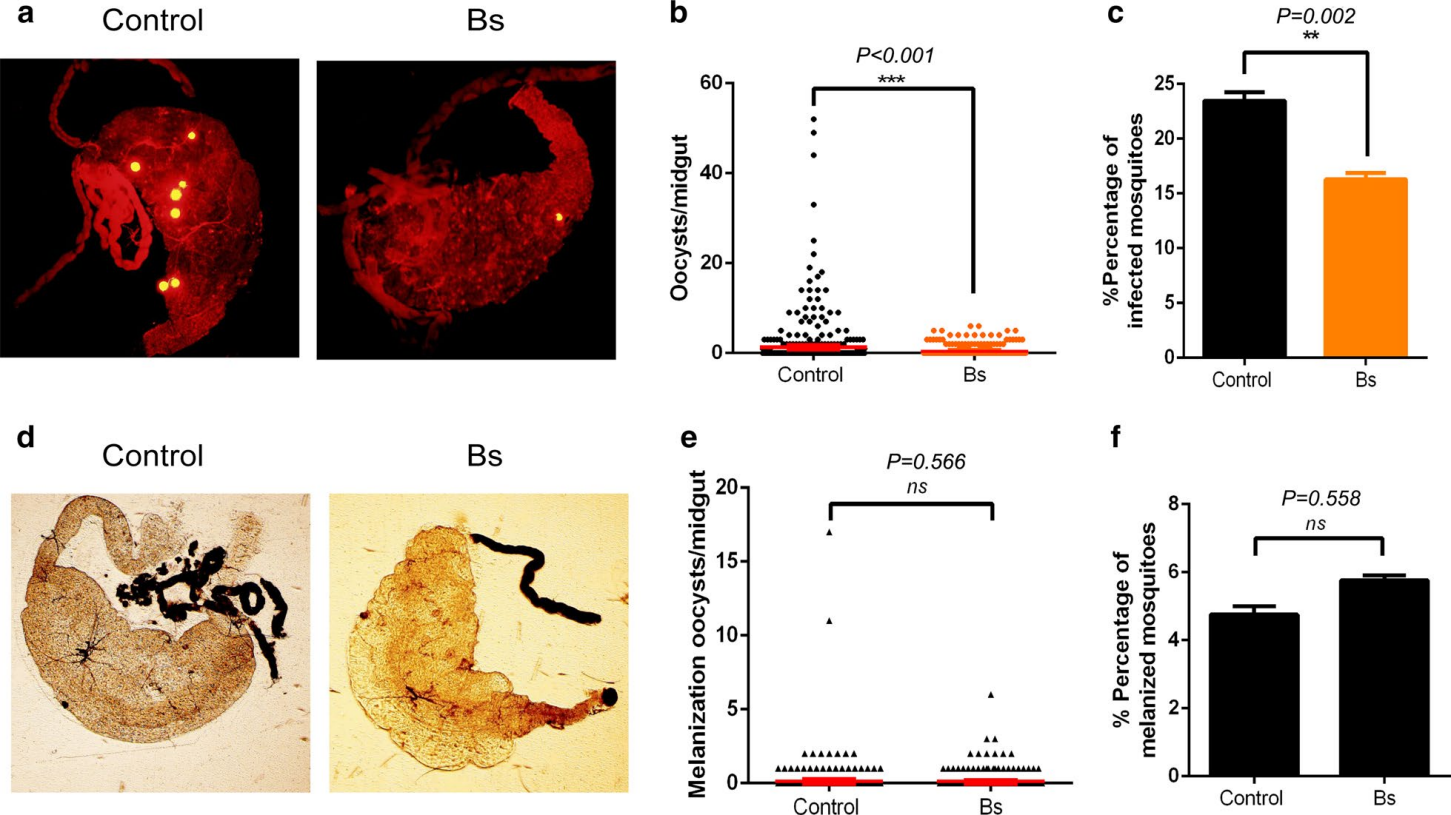
Bs exposure suppressed the susceptibility of *P. yoelii* of *An. dirus* without effect of melanization. **a** The infection of *P. yoelii* on midguts of *An. dirus* (yellow dots represent oocysts). **b** The infection intensities of *Plasmodium* on mosquito midguts in the control and Bs groups. **c** The infection rates of the control group and Bs group. **d** The melanized oocysts (black dots) on the midguts of mosquitoes. **e** The intensities of melanized *Plasmodium* on mosquito midguts in control and Bs groups. **f** The melanization rates in the two groups. *P < 0.05, **P < 0.01, ***P < 0.001; ns, non-significant

To investigate the impact of Bs exposure on the vector competence of *An. dirus* to *P. yoelii*, 523 mosquitoes from the control group and 536 mosquitoes from the Bs group were dissected and the oocysts were counted. The infection rates and intensities between control and Bs groups were compared. The infection intensity of the Bs group was significantly lower than that of the control group (Mann-Whitney U-test, *U* = 128711.000, *Z* = − 3.302, *P* ≤ 0.001) (Fig. 1a, b). The infection rates were 23.71% in the control group and 16.23% in the Bs group with a significant difference between the two groups (*χ*^2^ = 9.28, *df* = 1, *P *= 0.002) (Fig. 1c). Thus, the results implied that the vector competence of *An. dirus* was greatly decreased by a sublethal dose of Bs treatment.

There existed a melanization mechanism in *An. dirus*, as it was a non-suitable host to *P. yoelii* [21]. To test whether Bs treatment changes the vector competence of *An. dirus* to *P. yoelii* by affecting the melanization mechanism, the intensities and rates of melanization were compared between the control group and Bs group. As a result, no significant difference in melanized oocyst intensity was found between the two groups (Mann-Whitney U-test, *U *=  139046.500, *Z *= − 0.574, *P *= 0.566) (Fig. 1d, e). The rates of mosquitoes containing melanized oocysts were 4.97% and 5.78% in the control group and Bs group, respectively, also without significant difference (*χ*^2^= 0.343, *df *= 1, *P *= 0.558) (Fig. 1f). The data suggest that no melanization mechanism was involved in the decrease of vector competence of *An. dirus* to *P. yoelii*.

### Bs treatment elicits higher TEP1 expression for a longer time than that of the control

**Fig. 2:**
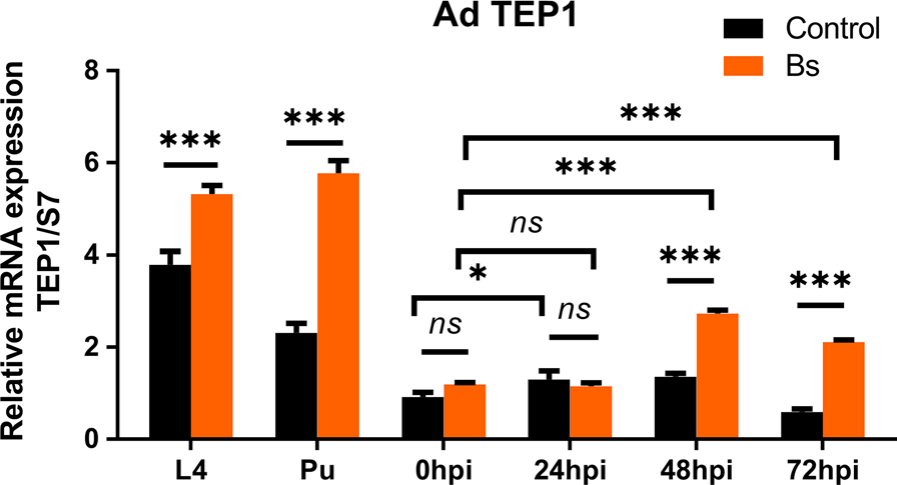
The elicitation of TEP1 expression following Bs treatment. The statistical significance of fold change values was determined *via* a t-test. *P < 0.05, **P < 0.01, ***P < 0.001; ns, non-significant. *Abbreviations*: L4, fourth-instar larvae; Pu, pupae; hpi, hours post-infection

The innate immune system of *Anopheles* was the main defense against parasites, bacteria, fungi and viruses, which was engaged at multiple stages of *Plasmodium* infection [22–24]. It was shown that the central mosquito complement component, thioester-containing protein 1 (TEP1), was strongly associated with protecting mosquitoes from infection by both rodent and human parasites [25–27]. To confirm whether the decrease of parasite load in *An. dirus* by Bs treatment was a result of regulating TEP1 expression, the transcriptional levels of the TEP1 gene at different stages and time points were assessed. The results showed that Bs treatment significantly increased TEP1 expression in the fourth-instar larvae (L4) and pupae (Pu), 48 h post-infection (hpi) and 72 hpi (*t *= 11.67, *df *= 24; *t *= 26.49, *df *= 24; *t *= 10.48, *df *= 24; *t* = 11.64, *df *= 24; *P* < 0.001 for all), which indicated that Bs treatment could activate the expression of TEP1 and improve the immune response of mosquitoes against *Plasmodium*. Moreover, TEP1 expression was elevated at 24 hpi and recovered below the basal level at 72 hpi in the control group. While in the Bs group, the TEP1 response to *Plasmodium* infection and upregulation by Bs exposure began at 48 hpi and lasted until 72 hpi (Fig. 2). These results demonstrated that Bs could not only upregulate the expression of TEP1, but also maintain the high expression of TEP1 for a longer time.

### Reduction of vector competence by Bs treatment was rescued by silencing TEP1 expression

**Fig. 3:**
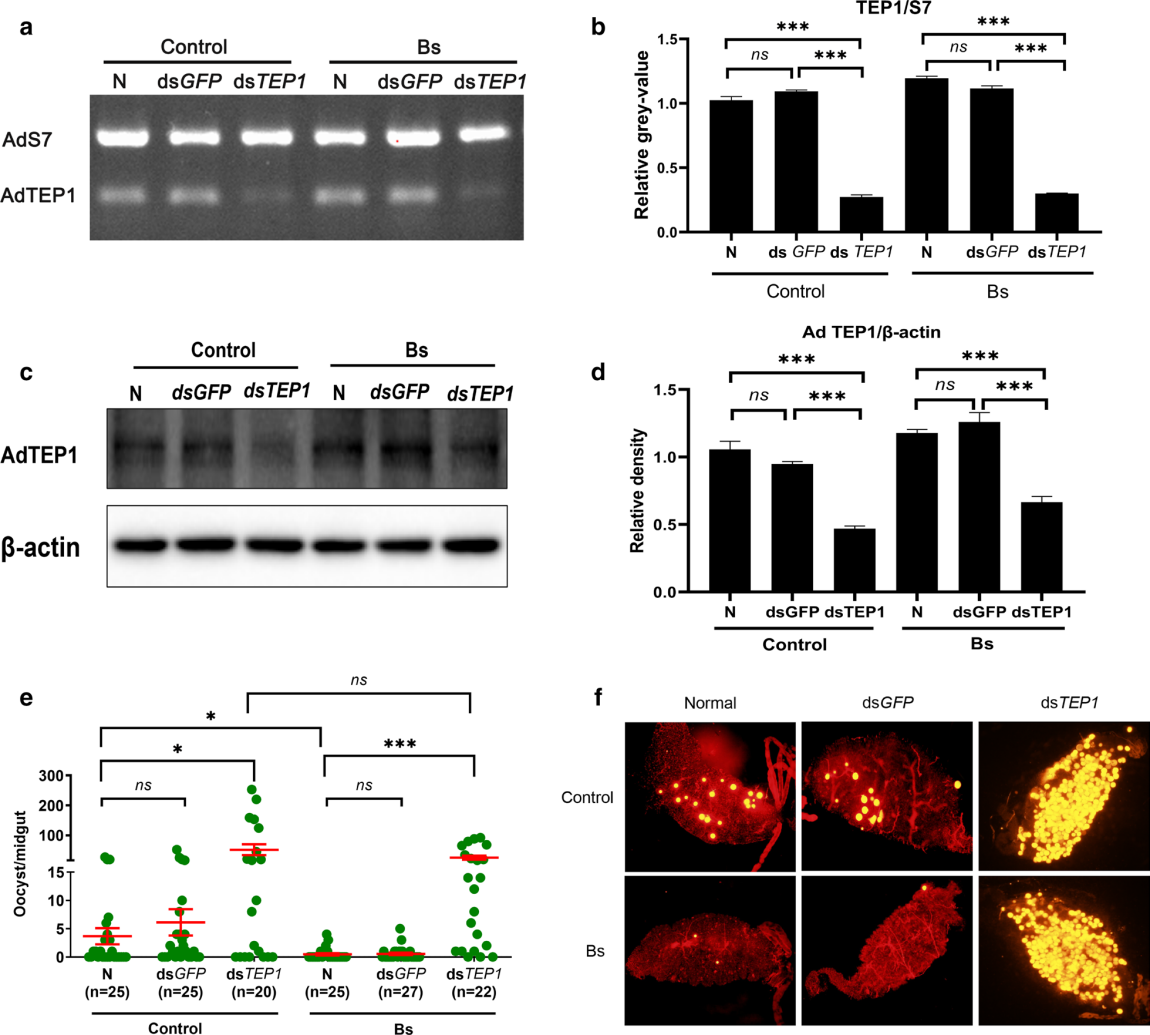
Depletion of AdTEP1 through RNAi gene silencing resulted in changes of *P. yoelii* oocyst intensity in Bs-treated and untreated mosquitoes. **a** Transcript variation of AdTEP1 in different groups after gene silencing by dsRNA. **b** The knockdown efficiencies of the control group and Bs group were 75.03% and 72.4%, respectively. **c** Three days post-dsRNA injection, western blot using AdTEP1-specific antibody showed that AdTEP1 protein was reduced in AdTEP1-dsRNA injection mosquitoes than that of GFP-dsRNA injection and normal mosquitoes both in the control and Bs groups. For each sample, total protein amount was determined by bicinchoninic acid assay, and an equal amount of protein was loaded for SDS-PAGE separation. Western blot with anti-actin antibody served as another loading control. **d** Three days post-dsRNA injection, the relative TEP1 protein expression in the control and Bs group decreased by 58.6% and 51.2%, respectively. **e** *Plasmodium yoelii* infection intensity (i.e. oocyst load) was increased in AdTEP1 dsRNA-treated mosquitoes both in control and Bs groups. No significant difference (*P *= 0.183) of oocyst loads was observed between the control TEP1 dsRNA-treated and Bs TEP1 dsRNA-treated groups. Points indicate the absolute value of parasite counts in individual mosquitoes. A Mann-Whitney U-test was used to determine the significance of differences in oocyst numbers. **f** Oocysts shown on the midguts of mosquitoes under a fluorescence microscope. *Abbreviations*: N, normal mosquitoes; dsGFP, GFP dsRNA-injected mosquitoes; dsTEP1, AdTEP1 dsRNA-injected mosquitoes

To further confirm whether TEP1 was involved in the reduction of vector competence by Bs treatment, a gene-silencing approach was carried out in Bs and control groups. The transcript knockdown efficiency was tested using semi-quantitative PCR and western-blot methods. As shown in Fig. 3a, PCR successfully amplified distinct TEP1 fragments in GFP dsRNA-injected and normal non-injected mosquitoes, but generated a weak product band in AdTEP1 dsRNA-injected mosquitoes either in the Bs or control groups, with silencing efficiencies of 75.03% and 72.4% in the control group and Bs group, respectively (Fig. 3b). Meanwhile, with AdTEP1 dsRNA injection, the protein expression in the control and Bs group decreased by 58.6% and 51.2%, respectively (Fig. 3c, d). When comparing between the normal mosquitoes and the GFP-dsRNA injected mosquitoes, the infection intensities were significantly increased by TEP1-dsRNA injection both in the control and Bs groups, which indicated that TEP1 plays a key role in the innate immunity of *An. dirus* against *P. yoelii* (Fig. 3e, f). However, no significant difference was found between TEP1-dsRNA injected mosquitoes from the control and Bs groups (Fig. 3e, f; Mann-Whitney U-test, *U *= 211.000, *Z *= − 0.228, *P *= 0.819), which suggested that Bs treatment could not suppress the development of *P. yoelii* in *An. dirus* in the absence of TEP1, and TEP1 played an important role in the inhibition of vector competence by Bs exposure.

### Imd signaling pathway was engaged in the elicitation of TEP1 by treatment of Bs

**Fig. 4:**
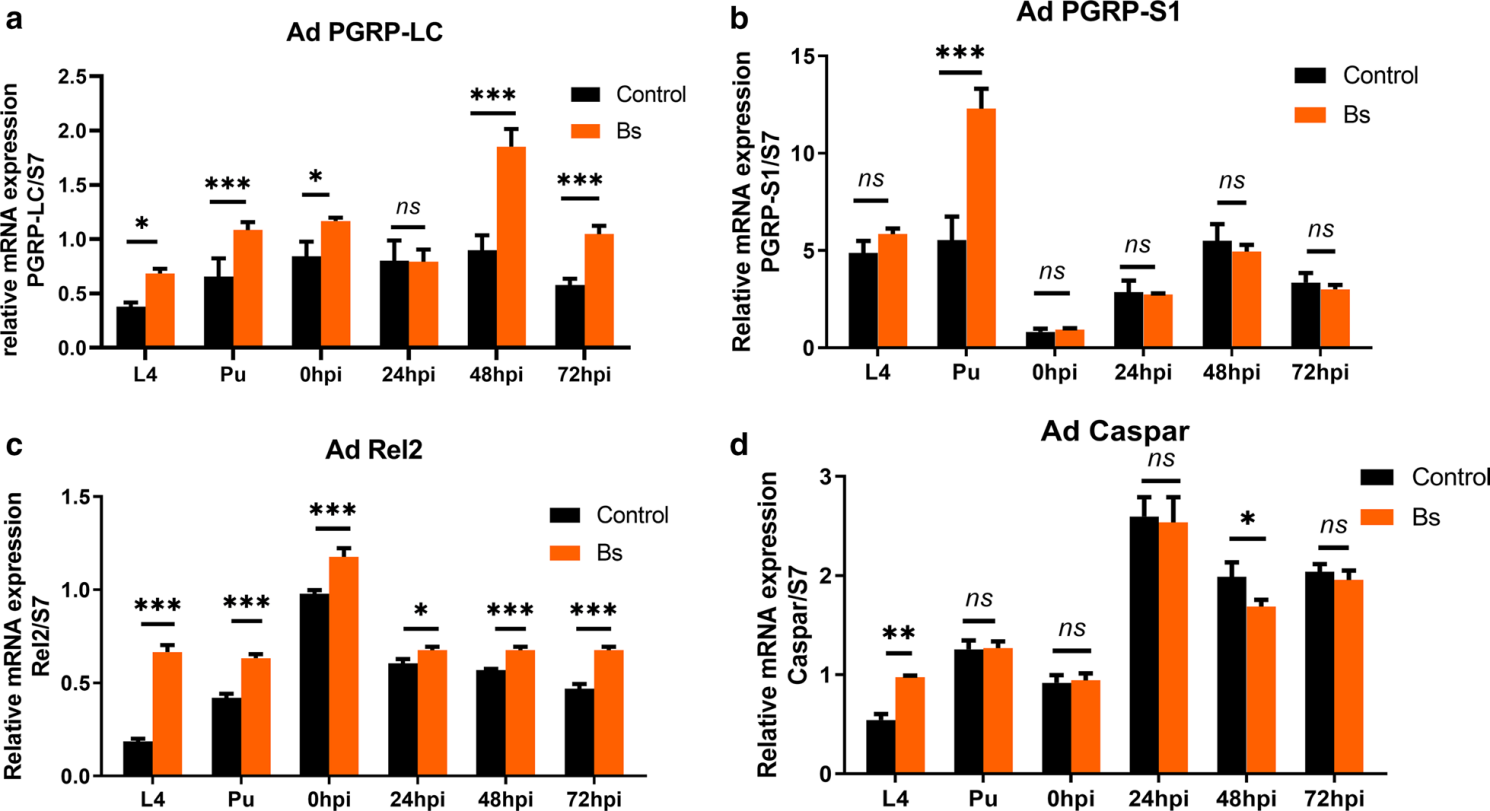
Elicitation of Imd pathway components expression with Bs treatment. Gene expression analysis of PGRP-LC (**a**), PGRP-S1 (**b**), Rel2 (**c**) and Caspar (**d**) of control and Bs-treated mosquitoes at L4, Pu, 0 hpi, 24 hpi, 48 hpi and 72 hpi. The statistical significance of fold change values was determined *via* a t-test. *P < 0.05, **P < 0.01, ***P < 0.001; ns, non-significant

To detect whether the Bs-mediated increase of TEP1 is achieved by activation of the Imd signaling pathway, we first evaluated the expression of the upstream components of the pathway such as the transmembrane long peptidoglycan recognition protein C (PGRP-LC) and the extracellular receptor PGRP-S1. Interestingly, the transcriptional level of PGRP-LC was significantly elevated by Bs treatment at most of the time points except 24 hpi (Fig. 4a). However, the expression of PGRP-S1 was only upregulated in pupae with no significant difference at other time points after Bs treatment (Fig. 4b). Furthermore, the expression of key transcription factor Relish 2 (Rel2) was also analyzed. A robust rise was observed in Bs-treated mosquitoes and this upregulation was maintained from larvae to 72 hpi (Fig. 4c). In contrast, the expression of the negative regulator Caspar, was decreased at 48 hpi after Bs treatment (only upregulated in the fourth-instar larvae (L4) (Fig. 4d). These results indicate that the Imd signaling pathway might be activated by Bs exposure and result in upregulation of TEP1 expression and a reduction of mosquito vector capacity.

## Discussion

*Bacillus sphaericus* is one of the most important biological agents, which can be used successfully against mosquito larvae [12, 14]. However, in actual application of Bs agents, a sublethal dose effect often exists owing to insufficient dosing. Therefore, it is very important to clarify whether the application of a sublethal dose Bs has an impact on the malaria transmission capacity of the surviving *Anopheles* mosquitoes. The extent to which Bs should be applied in the future should be given further consideration and assessment in case of possible unexpected negative effects, if Bs exposure could enhance the capacity of malaria transmission as we previously reported on *An. stephensi* which is a susceptible host of *P. yoelii* [28]. In the present study, we found that a sublethal dose of Bs treatment significantly reduced susceptibility of *An. dirus* to *P. yoelii*, with the decrease of *P. yoelii* infection intensity and rate. What is interesting is that Bs exposure showed contrary impact on vector competence of *An. dirus* and *An. stephensi* to a same pathogen *P. yoelii*. The different results from the susceptible and refractory animal models provide a warning that human malaria transmission under a susceptible model might be strengthened, which deserves further study. And, the underlying mechanisms of the difference might be due to the genetic background of various mosquito species and the symbiotic microflora in the guts of *An. stephensi* and *An. dirus*. To further explore the mechanisms, we are using high-throughput sequencing techniques to compare the transcriptome and gut flora of Bs-treated or untreated *An. stephensi* and *An. dirus* before and after infection by *P. yoelii*. Although the present result does not lead to an increase in malaria transmission, we do not recommend the use of sublethal doses of Bs, which can promote the emergence of drug resistance.

The mosquito midgut is the target organ where the toxin proteins of Bs come into play. Also, in the midgut, *Plasmodium* undergoes a series of complex developmental transitions and the majority of parasites are killed during this process. Although the phenomenon in this study is similar to “immune priming”, the possible mechanism of the vector competence reduction of *An. dirus* by Bs exposure may be different, as identical pathogens have been used for the attack in the “immune priming” study [16, 29], while two different pathogens including Bs and *Plasmodium* were used in the present study. To further explore the mechanisms of decreasing vector competence by Bs exposure, the mosquito’s innate immunity including melanization and the Imd signaling pathway was investigated in this study. Melanization is an insect innate immunity response typically associated with tissue damage and pathogen invasion [30, 31]. *Anopheles dirus* is refractory to *P. yoelii*, and there exists a melanization mechanism which plays a role in prophenoloxidase-mediated defense against malaria parasites [21]. In the present study, no significant difference of melanization rates and intensities was found between the control and Bs groups, which indicates that Bs does not reduce the vector competence by a melanization mechanism.

It is well known that the development of *Plasmodium* is mainly affected by mosquito innate immunity [32–34]. There are three major signaling pathways contributing to anti-*Plasmodium* defense, i.e. Toll, immune deficiency (Imd), and the Janus kinase-signal transducers and activators of transcription (JAK-STAT) pathways [35]. Research from Garver et al. [36] revealed that Thioester containing protein 1 (TEP1) was an effector molecule of the Imd signal pathway in anti-*Plasmodium* action. TEP1 was secreted by hemocytes and fat body, and bound to *Plasmodium* parasites leading to their death, which played an important role in the immunological function [37, 38]. Wang et al. [26] have found that TEP1 plays an important role in the resistance of *An. dirus* to *P. yoelii* by modulating the gut flora. In the present study, it was found that Bs exposure not only upregulated the basal expression of TEP1 in larval and pupal stages, but also lengthened TEP1 acting time. It was reported that TEP1 of *An. gambiae* started binding on the surface of ookinetes at 24 hpi, and the counts of TEP1-positive ookinetes increased with time and peaked at 48 hpi [27]. However, this study found that the expression of *An. dirus* TEP1 in the Bs group began to rise at 48 h post-infection by *P. yoelii* and kept a high level compared with the control group at least to 72 hpi, suggesting that Bs exposure can help the surviving mosquitoes to keep the killing action of *Plasmodium* for longer time. The RNAi results confirmed the role of TEP1 in the impact of Bs on vector competence of *An. dirus* to *P. yoelii*, as the increase in vector resistance disappeared after silencing of TEP1 expression.

In order to further verify whether the Imd signaling pathway was involved in the influence of Bs on vectorial capacity of *An. dirus*, several other key molecules in the Imd signaling pathway were investigated. PGRP-LC was known as the pattern recognition receptor (PRR) molecule, which activated the Imd pathway of mosquitoes in response to bacteria or *Plasmodium* infection [39]. Rel2 was an NF-κB transcription factor of the Imd pathway [35]. The activation of the Imd pathway could lead to the nuclear translocation of Rel2, and induced the expression of the immune genes [40]. As shown in the present study, the expression of PGRP-LC and Rel2 was obviously elevated by Bs exposure. Therefore, the data strongly supported that Bs exposure enhanced TEP1 expression through activating the Imd signal pathway and resulted in a reduction of vector competence of *An. dirus* to *P. yoelii*. The other two major innate immune pathways (Toll and JAK-STAT) were also assessed by expression analysis of their related genes, including MyD88, Tube, Rel1, STAT and PIAS2. Compared to the control group, no significant change of MyD88, Tube and PIAS2 genes’ expression was observed in Bs-treated mosquitoes (Additional file 2: Figure S1a, b, e). The expression of Rel1 and STAT changed irregularly by Bs treatment without significant difference between the control and Bs groups at 24 hpi and 48 hpi which were key periods of anti-*Plasmodium* action of *Anopheles* (Additional file 2: Figure S1c, d). The present data do not support the speculation that Toll and JAK-STAT pathways play role in the impact of Bs exposure on vector competence of *An. dirus* to *P. yoelii*, although they control immune attacks that limit the development of *P. berghei*, *P. falciparum*, and/or *P. vivax* [41–44].

The interactions and relationships of parasites, microbiota and mosquitoes are complex. As an exogenous microorganism, Bs may change the balance of microorganisms in the mosquito gut and affect the immune responses of mosquitoes against *Plasmodium*. The underlying mechanism of Bs affecting the Imd signaling pathway is an interesting issue. The mechanisms should be investigated in more depth in future.

## Conclusions

To the best of our knowledge, this study provides the first evidence showing that a sublethal dose of Bs significantly reduced the vector competence of *An. dirus* to malaria parasites, which revealed a new important role of Bs in addition to killing mosquito larvae. Meanwhile, we preliminarily found that the Imd signaling pathway was involved in the impact of Bs on vector competence of *An. dirus* by enhancing the expression of TEP1. Our data could not only help us to understand the impact and mechanism of Bs exposure on *Anopheles* vector competence to malaria, but also provide us with novel clues for wiping out malaria using vector control.

## Supplementary information

**Additional file 1: Table S1.** Primers used in the real-time PCR to examine transcriptional levels of genes in Toll and JAK-STAT pathways.

**Additional file 2: Figure S1.** Toll and JAK-STAT pathways were not obviously activated with Bs treatment. Gene expression analysis of Toll and JAK-STAT pathway components: MyD88 (**a**); Tube (**b**) and transcription factor REL1 (**c**); STAT (**d**); and PIAS2 (**e**) of control and Bs-treated mosquitoes at L4, Pu, 0 hpi, 24 hpi, 48 hpi and 72 hpi. The statistical significance of fold change values was determined *via* a t-test. *P < 0.05, **P < 0.01, ***P < 0.001. *Abbreviations*: ns, non-significant, L4, fourth-instar larvae; Pu, pupae; hpi, hours post-infection.

## Data Availability

All data generated or analyzed during this study are included in this published article and its additional files.

## Abbreviations

Bs: *Bacillus sphaericus*
SdBs: Sublethal dose of Bs
dsRNA: Double-stranded RNA
RNAi: RNA interference
TEP1: Thioester-containing protein 1
Imd pathway: The immune deficiency pathway
JAK-STAT: Janus kinase-signal transducers and activators of transcription
qPCR: Quantitative polymerase chain reaction
PGRP-LC: Long peptidoglycan recognition protein C
PRR: The pattern recognition receptor
Rel2: Relish 2
L4: Fourth-instar larvae
Pu: Pupae
hpi: Hours post-infection
