# Supplementary material for: Bacillus sphaericus exposure reduced vector competence of Anopheles dirus to Plasmodium yoelii by upregulating the Imd signaling pathway
Source: Parasit Vectors. 2020 Sep 5;13:446. doi: 10.1186/s13071-020-04321-w (PMC7487769; doi:10.1186/s13071-020-04321-w)
Supplement: Supplementary file 1 — Additional file 1: Table S1. Primers used in the real-time PCR to examine transcriptional levels of genes in Toll and JAK-STAT pathways. [file 13071_2020_4321_MOESM1_ESM.docx]

**Additional file 1: Table S1.** Primers used in Real-time PCR to examine transcriptional levels of genes in Toll and JAK-STAT pathways

| Gene | Forward primer (5’-3’) | Reverse primer (5’-3’) |
| --- | --- | --- |
| **Ad MyD88** | ACGGGATTTGGTGGCTGAACTTC | GCGGGTTGGGTTGGGATTAGTG |
| **Ad Tube** | TGGACGGGACGAGAGTGTGAAG | TTCGGTTCGTGCTGCTGGAATG |
| **Ad Rel1** | GGTAATCGGTGGTCCCATCTCTCC | GGTTGCTGCTCTGGTGTAGTAGTG |
| **Ad STAT** | AGGCGGAGGACTATCTGCTCAAG | GCGGTGAACGGCTGGATGTG |
| **Ad PIAS2** | GTTGAAGCACCGAGTCCGAGAAC | CGTCTGTTCCACCGCACTACTG |
