# Supplementary material for: Bacillus sphaericus exposure reduced vector competence of Anopheles dirus to Plasmodium yoelii by upregulating the Imd signaling pathway
Source: Parasit Vectors. 2020 Sep 5;13:446. doi: 10.1186/s13071-020-04321-w (PMC7487769; doi:10.1186/s13071-020-04321-w)
Supplement: Supplementary file 2 — Additional file 2: Figure S1. Toll and JAK-STAT pathways were not obviously activated with Bs treatment. Gene expression analysis of Toll and JAK-STAT pathway components: MyD88 (a); Tube (b) and transcription factor REL1 (c); STAT (d); and PIAS2 (e) of control and Bs-treated mosquitoes at L4, Pu, 0 hpi, 24 hpi, 48 hpi and 72 hpi. The statistical significance of fold change values was determined via a t-test. *P < 0.05, **P < 0.01, ***P < 0.001. Abbreviations: ns, non-significant, L4, fourth-instar larvae; Pu, pupae; hpi, hours post-infection. [file 13071_2020_4321_MOESM2_ESM.docx]

**
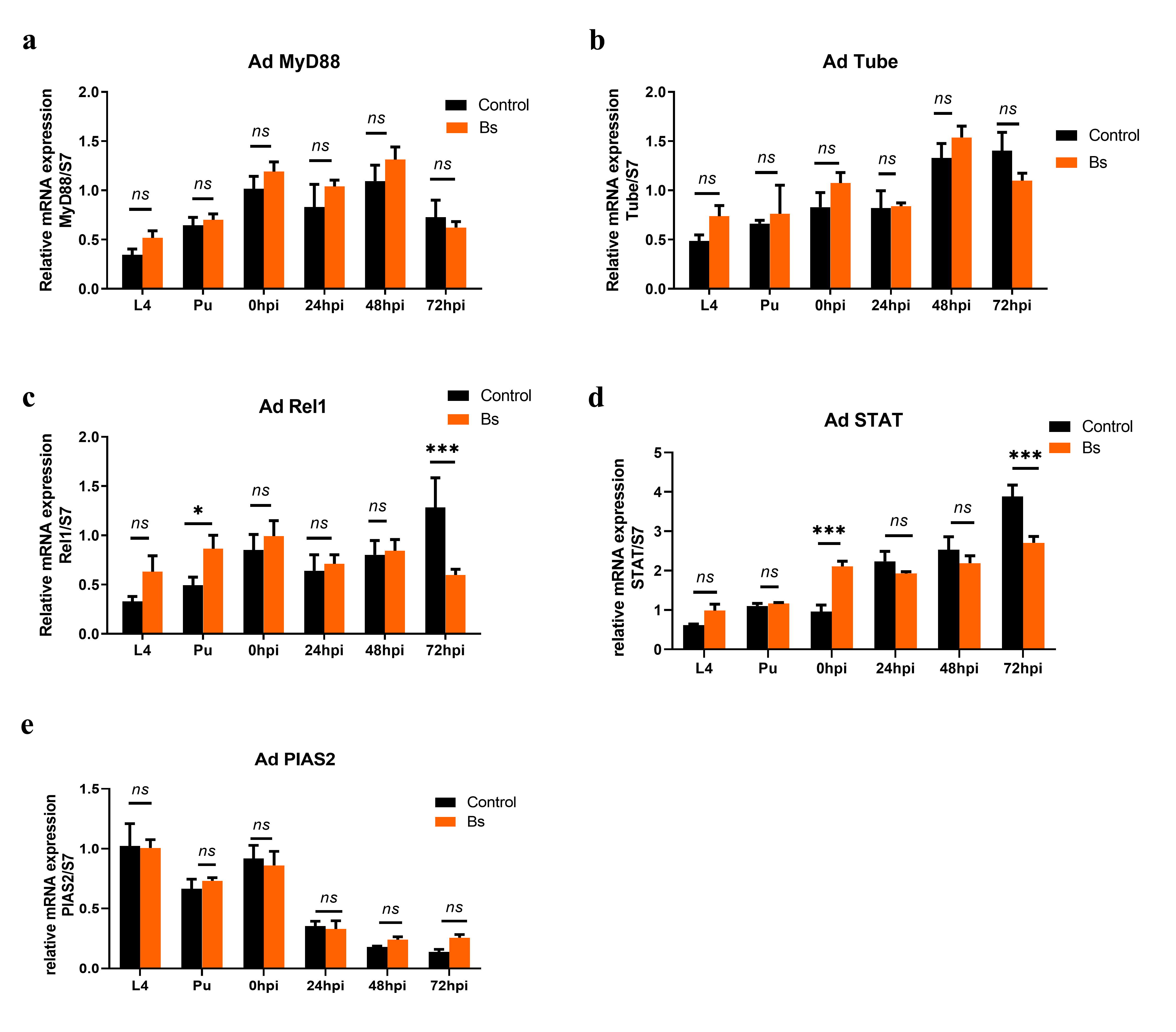
**

**Additional file 2: Figure S1.** Toll and JAK-STAT pathways were not obviously activated with Bs treatment. Gene expression analysis of Toll and JAK-STAT pathway components: MyD88 (**a**); Tube (**b**) and transcription factor REL1 (**c**); STAT (**d**); and PIAS2 (**e**) of control and Bs-treated mosquitoes at L4, Pu, 0 hpi, 24 hpi, 48 hpi and 72 hpi. The statistical significance of fold change values was determined *via* a t-test. **P* < 0.05, ***P* < 0.01, ****P* < 0.001. *Abbreviations*: ns, not significant, L4, fourth-instar larvae; Pu, pupae; hpi, hours post-infection.
